# Triac Treatment Prevents Neurodevelopmental and Locomotor Impairments in Thyroid Hormone Transporter Mct8/Oatp1c1 Deficient Mice

**DOI:** 10.3390/ijms24043452

**Published:** 2023-02-09

**Authors:** Jiesi Chen, Eva Salveridou, Lutz Liebmann, Sivaraj M. Sundaram, Denica Doycheva, Boyka Markova, Christian A. Hübner, Anita Boelen, W. Edward Visser, Heike Heuer, Steffen Mayerl

**Affiliations:** 1 Leibniz Institute on Aging/Fritz Lipmann Institute, 07745 Jena, Germany; 2 Leibniz Research Institute for Environmental Medicine, 40225 Düsseldorf, Germany; 3 Department of Endocrinology, Diabetes & Metabolism, University Hospital Essen, University Duisburg-Essen, 45147 Essen, Germany; 4 Institute for Human Genetics, University Hospital Jena, Friedrich-Schiller University, 07747 Jena, Germany; 5 Endocrinology Laboratory, Department of Clinical Chemistry, Amsterdam Gastroenterology, Endocrinology & Metabolism, Academic Medical Center (AMC), 1105 AZ Amsterdam, The Netherlands; 6 Academic Centre for Thyroid Diseases, Department of Internal Medicine, Erasmus University Medical Center Rotterdam, 3015 GD Rotterdam, The Netherlands

**Keywords:** Allan-Herndon-Dudley syndrome, thyroid hormone transport, SLC16A2, SLCO1C1, thyroid hormone analog, Ditpa, Triac

## Abstract

Patients with inactive thyroid hormone (TH) transporter MCT8 display intellectual disability due to compromised central TH transport and action. As a therapeutic strategy, application of thyromimetic, MCT8-independent compounds Triac (3,5,3′-triiodothyroacetic acid), and Ditpa (3,5-diiodo-thyropropionic acid) was proposed. Here, we directly compared their thyromimetic potential in Mct8/Oatp1c1 double knock-out mice (Dko) modeling human MCT8 deficiency. Dko mice received either Triac (50 ng/g or 400 ng/g) or Ditpa (400 ng/g or 4000 ng/g) daily during the first three postnatal weeks. Saline-injected Wt and Dko mice served as controls. A second cohort of Dko mice received Triac (400 ng/g) daily between postnatal weeks 3 and 6. Thyromimetic effects were assessed at different postnatal stages by immunofluorescence, ISH, qPCR, electrophysiological recordings, and behavior tests. Triac treatment (400 ng/g) induced normalized myelination, cortical GABAergic interneuron differentiation, electrophysiological parameters, and locomotor performance only when administered during the first three postnatal weeks. Ditpa (4000 ng/g) application to Dko mice during the first three postnatal weeks resulted in normal myelination and cerebellar development but only mildly improved neuronal parameters and locomotor function. Together, Triac is highly-effective and more efficient than Ditpa in promoting CNS maturation and function in Dko mice yet needs to be initiated directly after birth for the most beneficial effects.

## 1. Introduction

Patients carrying inactivating mutations in the thyroid hormone (TH)-specific monocarboxylate transporter 8 (MCT8) show global developmental delays and a complex cluster of severe intellectual and motor disabilities (Allan-Herndon-Dudley syndrome (AHDS)) [1,2,3,4]. Additionally, patients exhibit characteristic changes in the TH serum profile with highly elevated T3 in the presence of normal/low T4 and normal/elevated TSH. According to the prevailing hypothesis, the neurological impairments of AHDS patients are caused by an insufficient TH transport into the brain while peripheral tissues sense the elevated serum T3 concentrations and are, therefore, in a hyperthyroid state [4,5,6]. That the CNS of MCT8 patients is indeed in a TH deficient state could be substantiated by histopathological findings that include hypomyelination, decreased cerebral expression of parvalbumin (PV), a calcium-binding protein present in a distinct subset of inhibitory neurons, and a delayed cerebellar development [6,7]. Tissue-specific alterations in TH content are also a characteristic feature of Mct8 ko mice that fully replicate the abnormal serum TH profile and show increased TH concentrations in peripheral tissues. Mct8 ko mice also exhibit a diminished passage of T3 into the CNS but are still capable of transporting T4 into the CNS [8,9,10]. This latter observation can be explained by the presence of the T4-specific transporter Oatp1c1 in murine blood-brain barrier cells [11,12]. Indeed, only Mct8/Oatp1c1 double knock-out mice (Dko) show a strongly diminished TH content in the CNS, similar morphological alterations, and disturbed neural differentiation as seen in patients, as well as profound locomotor impairments [13]. Considering these similarities, Dko mice represent a suitable mouse model for AHDS and are, therefore, a valuable tool for testing therapeutic interventions.

One of the most promising therapeutic approaches is the application of TH analogs such as 3,5-diiodo-thyropropionic acid (Ditpa) and 3,5,3′-triiodothyroacetic acid (Triac) that activate TH receptors and that do not depend on MCT8 for cellular entry [14,15,16,17]. Studies in Mct8-deficient mice and AHDS patients have already revealed that both compounds are capable of lowering endogenous TH production and normalizing symptoms of a peripheral thyrotoxicosis (such as hypermetabolism, muscle wasting, and increased heart rate) [18,19,20,21]. It is, however, still a matter of debate to which extent and during which time-period both compounds can exert beneficial effects on brain maturation and function.

Here, we provide the first direct comparison of Triac versus Ditpa action on brain parameters in Dko mice. Our results clearly indicate that Triac is more effective than Ditpa in promoting a normal brain development and maturation. Moreover, our studies revealed that the most beneficial effects are obtained if Triac treatment is initiated directly after birth while a delayed treatment onset significantly compromises the efficacy of the TH analog.

## 2. Results

### 2.1. Comparison of Triac versus Ditpa Treatment during Early Postnatal Development

In a pilot study, we demonstrated that treatment of Dko mice between postnatal day 1 (P1) and P11 with Triac at a concentration of 50 ng/g body weight (bw) (T (50)) or 400 ng/g bw (T (400)) resulted in a dose-dependent improvement of Purkinje cell (PC) dendritogenesis and myelination in the cerebral cortex at P12 [16]. To directly compare the thyromimetic effects of Triac and Ditpa, we expanded the design of this study and injected newborn Dko animals during the same postnatal period with either Triac (T (50) or T (400)) as previously reported [16] or with Ditpa at either 400 ng/g bw (D (400)) or 4000 ng/g bw (D (4000)).

**Figure 1:**
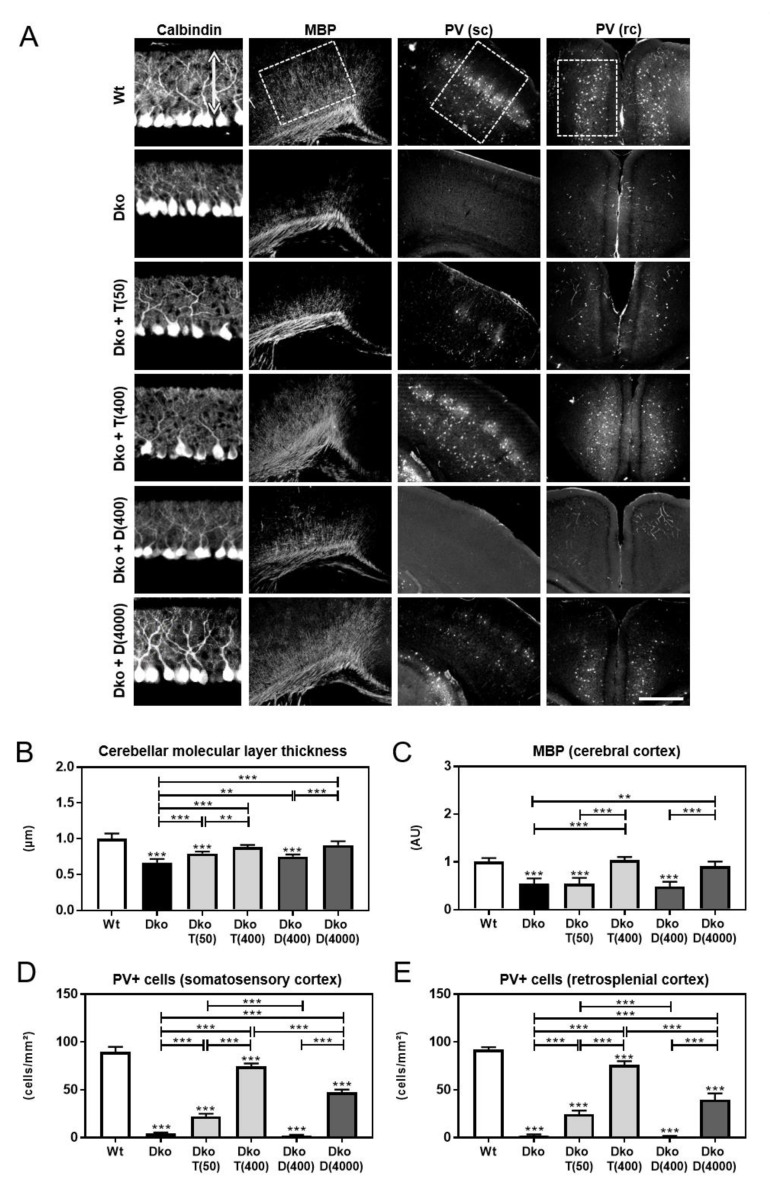
TH-analog treatment initiated after birth improves brain development. Newborn mice received a daily injection of saline (control) or different doses (in ng/g bw) of Triac or Ditpa between postnatal days 1 (P1) and P11, as indicated. (**A**) At P12, cerebellar Purkinje cell development was monitored by calbindin immunostaining and myelination in the cerebral cortex by MBP immunoreactivity, and PV+ interneurons were visualized in the somatosensory cortex (sc) and retrosplenial cortex (rc). (**B**) Dimension of the Purkinje cell dendritic tree (arrow in (**A**)) was measured highlighting a reduced thickness in Dko animals that was restored dose-dependently by Triac and Ditpa treatment. (**C**) MBP integrated signal density was determined in the inner layers of the cerebral cortex (box in (**A**)) showing reduced levels in Dko animals that normalized only upon high-dose Triac or Ditpa administration. PV+ interneurons were enumerated in the sc (**D**) and rc (**E**) (boxes in (**A**)). PV+ cells were almost absent in Dko mice and increased dose-dependently following Triac or high-dose Ditpa application. n = 3–5. Scale bars 50 µm (Calbindin), 500 µm (MBP), 250 µm (PV). **, *p* < 0.01; ***, *p* < 0.001.

Visualizing cerebellar PC dendritogenesis by calbindin immunofluorescence staining confirmed a strongly reduced dendritic outgrowth in Dko mice and, consequently, a much smaller molecular layer that reached only 60% of the thickness found in Wt animals at P12 (Figure 1A,B). Application of high doses of Triac or Ditpa to Dko mice fully restored PC outgrowth while the low dose applications were less effective. Analysis of myelin basic protein (MBP) immunoreactivity in the cerebral cortex revealed similar findings. Only in Dko mice treated with Triac or Ditpa at a high dose could normal MBP immunoreactivity be achieved (Figure 1A,C). As another indicator for thyromimetic action in the CNS, we analyzed the number of GABAergic interneurons expressing the calcium-binding protein parvalbumin (PV) in the primary somatosensory cortex as well as the retrosplenial cortex. In agreement with previous data [16], Dko mice exhibited a highly reduced number of PV+ cells in both regions (Figure 1A,D,E). High-dose Triac treatment restored 80% of the PV+ interneuron population in both areas whereas high-dose Ditpa treatment improved PV+ cell numbers only to 50% of the respective Wt values.

**Figure 2:**
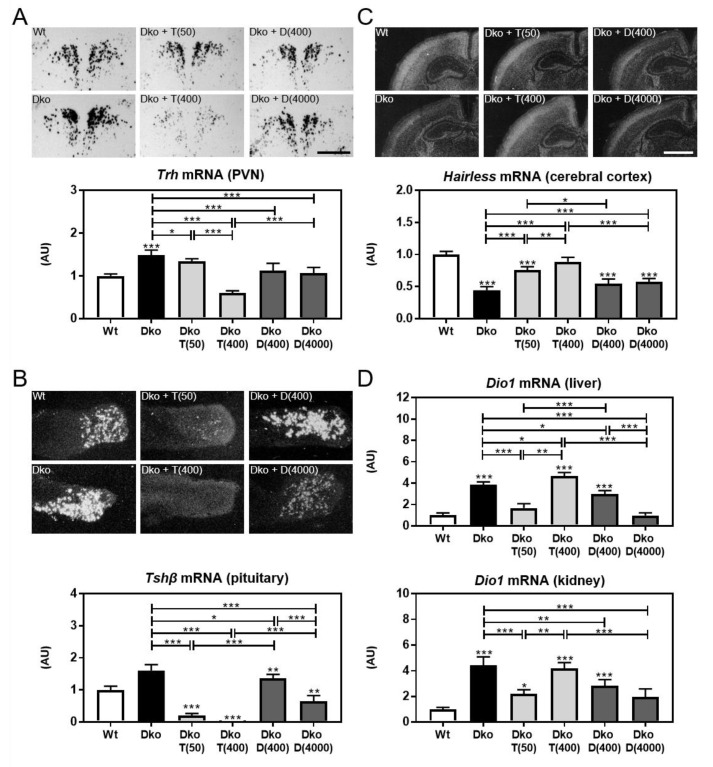
Triac executes stronger thyromimetic actions on the HPT axis than Ditpa. Dko mice were injected daily with either a saline solution or different concentrations of Triac and Ditpa between P1 and P20. At P21, activity of the HPT axis was monitored. (**A**) Saline-treated Dko mice exhibited strongly increased *Trh*-specific ISH signals in PVN neurons, which were reduced in intensity following Triac application in a dose-dependent manner. Ditpa had only little effect on *Trh* expression. Bright-field images are depicted while dark-field illuminations were employed for quantification. (**B**) Dark-field autoradiograms illustrate alterations in *Tshb* transcripts in the pituitary with increased levels in Dko mice that were almost completely suppressed by Triac application, but only moderately so by Ditpa treatment. (**C**) *Hr*-specific hybridization signals were used to examine thyromimetic effects of TH analogs in the brain. Centrally severely hypothyroid Dko mice present with strongly decreased hairless expression that was restored by Ditpa and, even more efficiently, by Triac application. (**D**) Hepatic and renal *Dio1* expression were studied by qPCR. Mice receiving the low dose of Triac showed a reduction in *Dio1* expression in both organs while high-dose Triac maintained elevated *Dio1* transcript levels as seen in saline-treated Dko mice. Ditpa decreased *Dio1* expression dose-dependently. n = 4–10. Scale bars 200 µm (*Trh*), 500 µm (*Tshb*), 1 mm (*Hairless*). *, *p* < 0.05; **, *p* < 0.01; ***, *p* < 0.001.

Application of TH analogs will affect the activity of the hypothalamus-pituitary-thyroid (HPT) axis by downregulating thyrotropin-releasing hormone (Trh) and thyroid- stimulating hormone subunit beta (Tshb) expression. To directly compare Triac versus Ditpa effects, we injected Dko mice between P1 and P20 with the same low and high doses paradigm as above and sacrificed the animals at P21. Radioactive in situ hybridization (ISH) studies on brain and pituitary sections confirmed elevated *Trh* mRNA expression in hypothalamic paraventricular nucleus (PVN) neurons and increased *Tshb* mRNA expression in the anterior pituitary in Dko mice (Figure 2A,B). Only high-dose Triac treatment reduced *Trh* expression whereas both Triac concentrations caused a profound downregulation of pituitary *Tshb* mRNA expression. In response to Ditpa treatment, Dko mice only showed a mild reduction in *Trh* transcript expression independent of the injected dose and a moderate decrease in *Tshb* specific expression signals upon high-dose Ditpa application. These data indicate that Triac exerts stronger thyromimetic action than Ditpa on the HPT axis.

We further assessed the expression of TH-sensitive markers in different organs to evaluate the respective tissue-specific thyroidal state. In the CNS, we analyzed mRNA expression of hairless (*Hr*), a gene well-established to be positively regulated by TH, by ISH (Figure 2C). *Hr*-specific signal intensities were strongly reduced in the cerebral cortex of Dko mice and increased dose-dependently following Triac treatment whereas Ditpa had only little stimulating effect on *Hr* mRNA expression in the CNS. In liver and kidney, increased deiodinase type 1 (*Dio1)* expression (Figure 2D) confirmed a hyperthyroid state of these tissues in Dko mice [13]. Low-dose Triac treatment significantly reduced *Dio1* transcript levels in both organs whereas Dko animals treated with the high dose of Triac showed similarly elevated *Dio1* levels as found in Dko mice. Low-dose Ditpa treatment caused slightly reduced renal and hepatic *Dio1* expression while application of high-dose Ditpa resulted in normal hepatic *Dio1* levels and slightly elevated renal *Dio1* expression. Altogether, these results suggest that Triac exerts a much stronger thyromimetic effect than Ditpa in all analyzed organs.

### 2.2. Locomotor Behavior of Triac versus Ditpa Treated Dko Animals

Next, we addressed the question of whether a postnatal treatment with TH analogs had any beneficial effect on locomotion. To this end, Dko mice were injected with either a high dose of Triac (400 ng/g bw) or Ditpa (4000 ng/g bw) for the first three postnatal weeks. Thereafter, TH-analog treatment was terminated, and adult animals at the age of 7–9 weeks were subjected to locomotor tests.

**Figure 3:**
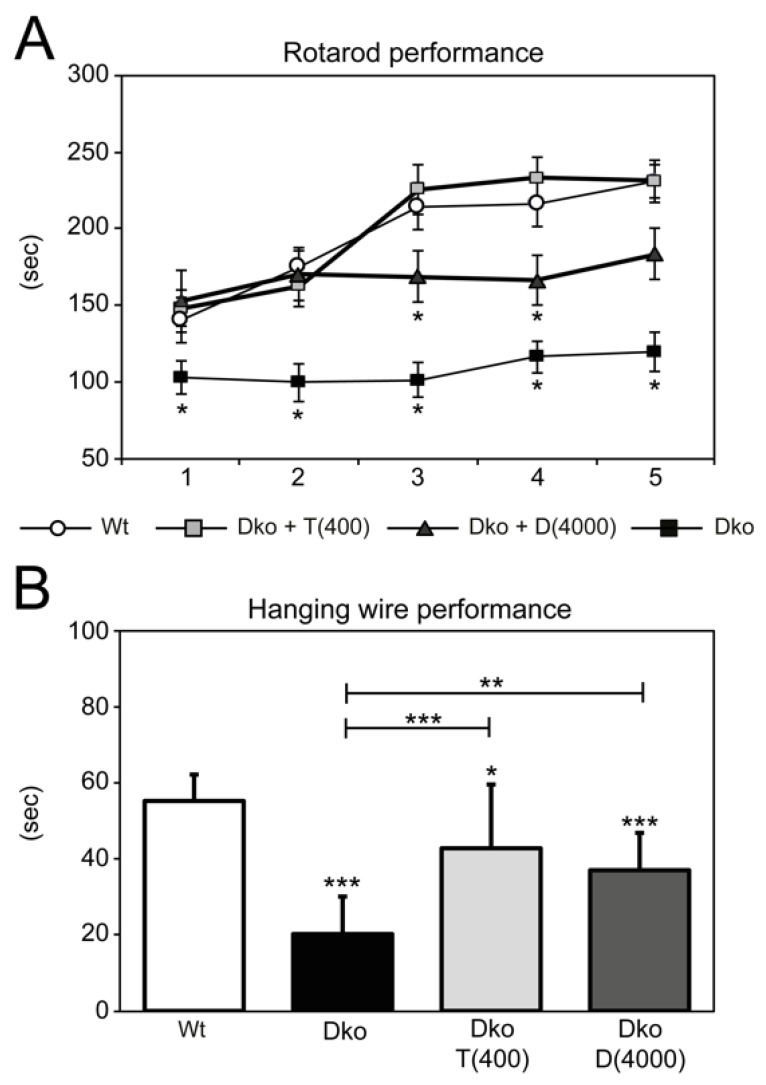
Transient early TH-analog treatment improves locomotion in adulthood. Dko mice received daily injections with saline, 400 ng/g bw Triac (Dko + T (400)) or 4000 ng/g bw Ditpa (Dko + D (4000)) between P1 and P20. Thereafter, treatment was ceased, and locomotor behavior was addressed at the age of 7–9 weeks (**A**) In contrast to Dko mice receiving saline and showing severe locomotor deficiencies, Ditpa application moderately and Triac treatment fully normalized performance on an accelerating rotarod. (**B**) Neuromuscular abnormalities were further examined by a hanging wire test. Dko mice clung to the wire for only a short time-period, which was significantly improved in TH analog-treated experimental groups. n = 10–14. *, *p* < 0.05; **, *p* < 0.01; ***, *p* < 0.001 significant difference to Wt and Dko + T (400) mice.

In accordance with previous data [13], Dko mice spent significantly shorter time on the accelerating rotarod compared to control mice and did not display any visible improvement in their performance during the 5 days of the training period (Figure 3A). In contrast, Triac-treated Dko mice stayed on the rotating wheel as long as Wt mice and significantly improved their skills during the training period. The performance of Ditpa-treated Dko mice was initially not significantly different from that of Wt mice. However, these animals failed to further improve their riding time during the 5-day testing period, pointing to motor-learning deficits. Subsequently, animals were subjected to a hanging wire test for muscle strength assessment (Figure 3B). Wt animals could easily cling on the metal wire for 60 s whereas Dko animals fell off after 20 s. Both Triac and Ditpa treatment significantly increased the hanging time of Dko mice.

### 2.3. Long-Term Effects of Triac versus Ditpa on the HPT Axis and Brain Morphology

Following locomotor assessment, animals were sacrificed at the age of 10 weeks, and the activity of the HPT axis was evaluated by analyzing *Trh* and *Tshb* transcript levels by ISH and determining serum TH values. At this time point, animals have not been injected with TH analogs for 7 weeks, and, therefore, a reconstitution of the abnormal HPT axis parameters in all Dko animals could be envisioned. Hypothalamic *Trh* mRNA expression as assessed by ISH (Figure S1A) was equally high and serum T4 levels were equally low (Figure S1B) in all Dko mice independent of their treatment during the first 3 postnatal weeks. Serum T3 concentrations were elevated in Ditpa- and Triac-treated Dko mice, but to a significantly lesser extent than in Dko animals (Figure S1B). Likewise, TH-analog treated Dko animals showed lower *Tshb* expression than Dko mice (Figure S1A). These findings suggest that early postnatal treatment with TH analogs induces long-lasting changes in the set point of the HPT axis in Dko animals.

**Figure 4:**
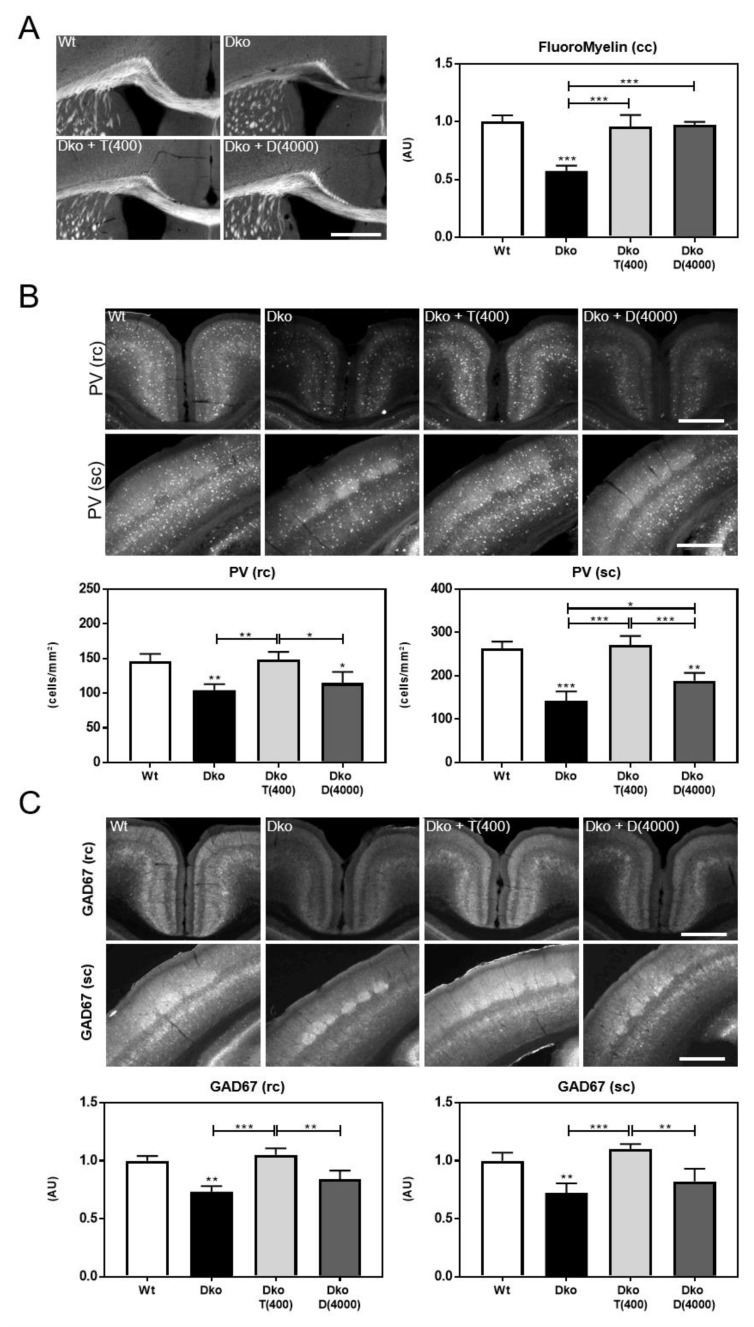
Long-term morphological alterations are induced by early postnatal TH-analog application. Following transient treatment with TH analogs Triac and Ditpa (in ng/g bw) between P1 and P20, brain parameters were examined at 10 weeks of age. (**A**) Myelination was evaluated in the corpus callosum (cc) by FluoroMyelin staining. TH-analog treatment could rescue the hypomyelination phenotype of Dko mice. (**B**) PV+ interneurons were visualized in the retrosplenial cortex (rc) and somatosensory cortex (sc). PV cell numbers were low in saline injected Dko animals, but fully restored in both areas upon Triac treatment. Ditpa only mildly improved PV+ numbers in the sc. (**C**) GAD67 immuno-reactivity analyzed in the same cortical regions demonstrated reduced integrated densities in saline injected Dko mice that was unaffected by Ditpa, but fully restored by Triac application. n = 3–4. Scale bars 500 µm (FluoroMyelin), 250 µm (PV, GAD67). *, *p* < 0.05; **, *p* < 0.01; ***, *p* < 0.001.

In addition, we investigated the long-term effects of early postnatal TH-analog treatment on brain morphology. To examine CNS myelination, coronal vibratome sections were subjected to FluoroMyelin staining and fluorescence intensities were quantified in the corpus callosum area (Figure 4A). Compared to control animals, Dko mice showed strongly reduced myelin content. Triac- and Ditpa-treated Dko mice exhibited similar FluoroMyelin staining intensities as Wt animals suggesting a normalization of myelin formation. In order to assess the maturation state of PV-expressing GABAergic neurons in retrosplenial and somatosensory cortex, PV+ cells were visualized by immunofluorescence staining. In contrast to Dko mice, Triac-treated Dko animals showed similar numbers of PV+ cells in both areas as found in Wt mice (Figure 4B). Interestingly, Ditpa treatment only slightly increased PV+ cell numbers in the somatosensory cortex but did not exert any beneficial effect in the retrosplenial cortex. As PV is present only in a subset of GABAergic neurons, we additionally investigated expression of glutamate decarboxylase 67 (GAD67) as a key marker for all GABA-producing interneurons. Quantification of GAD67 immunoreactivity in the somatosensory and retrosplenial cortex showed significantly reduced values in saline treated Dko mice (Figure 4C). Importantly, application of Triac, but not Ditpa, restored normal GAD67 expression in Dko mice. Altogether, our data demonstrate that a transient treatment of Dko mice with TH analogs during early postnatal stages induces long-lasting morphological changes in the CNS with Triac being more effective than Ditpa in normalizing brain parameters of Dko animals.

### 2.4. Electrophysiological Studies

**Figure 5:**
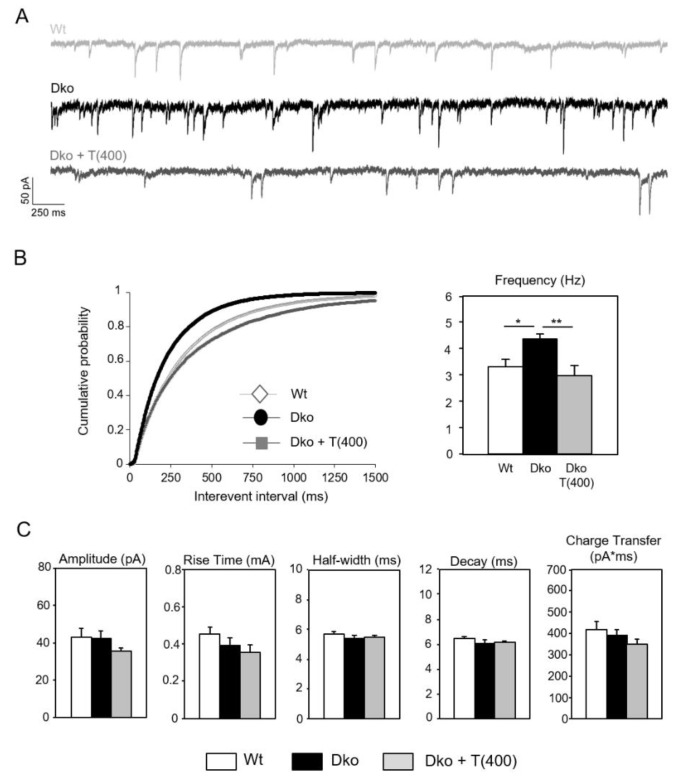
Deletion of Mct8/Oatp1c1 modulates GABAergic transmission in the cortex. Electrophysiological parameters were recorded on coronal forebrain sections using patch-clamp technique. (**A**) Representative traces of mIPSCs of Wt, Dko, and Dko + T (400) recorded from pyramidal neurons of the somatosensory cortex. (**B**) Cumulative plot and bar chart of mIPSC frequencies demonstrating a significant increase in Dko animals that can be rescued by application of Triac for 3 weeks. (**C**) Amplitude, rise time, half-width, time constant of decay, and transported electric charges are not modified in neurons from Dko animals. n = 17–18. *, *p* < 0.05; **, *p* < 0.01.

Low GAD67 expression may result in diminished local GABA production and inhibition in the cerebral cortex that, in turn, will greatly influence cortical network activity. Therefore, we assessed GABAergic synaptic transmission in the somatosensory cortex in slices obtained from Wt, untreated Dko, and Dko mice treated with high-dose Triac for the first three postnatal weeks. Patch-clamp recordings were performed, and spontaneous miniature inhibitory postsynaptic current (mIPSC) kinetics were analyzed. Representative traces recorded from pyramidal neurons in cortical layers II/III are shown in Figure 5A. No differences in passive membrane properties (capacity: Wt 43.7 ± 4.3 pF, Dko 36.8 ± 1.8 pF; Dko + T (400) 35.8 ± 2.0 pF and input resistance: Wt 67.0 ± 4.6 MΩ; Dko 69.3 ± 6.4 MΩ; Dko + T (400) 65.2 ± 6.8 MΩ) were observed. In contrast, mean frequencies of mIPSCs recorded from pyramidal neurons of the somatosensory cortex were significantly increased in Dko slices compared to Wt slices (Figure 5B) suggesting that Mct8/Oatp1c1 inactivation has a major effect on GABAergic transmission in the cerebral cortex. Other recorded mIPSC kinetic parameters such as amplitudes, rise time, half-width, time constant of decay, and transported electric charges did not differ between the genotypes (Figure 5C). Interestingly, Triac-treated Dko mice showed fully normalized mIPSC frequencies. This observation underscores the prominent and beneficial effects of Triac treatment on the development and function of the inhibitory system in Dko mice.

### 2.5. Determining the Critical Time Window of Triac Action

**Figure 6:**
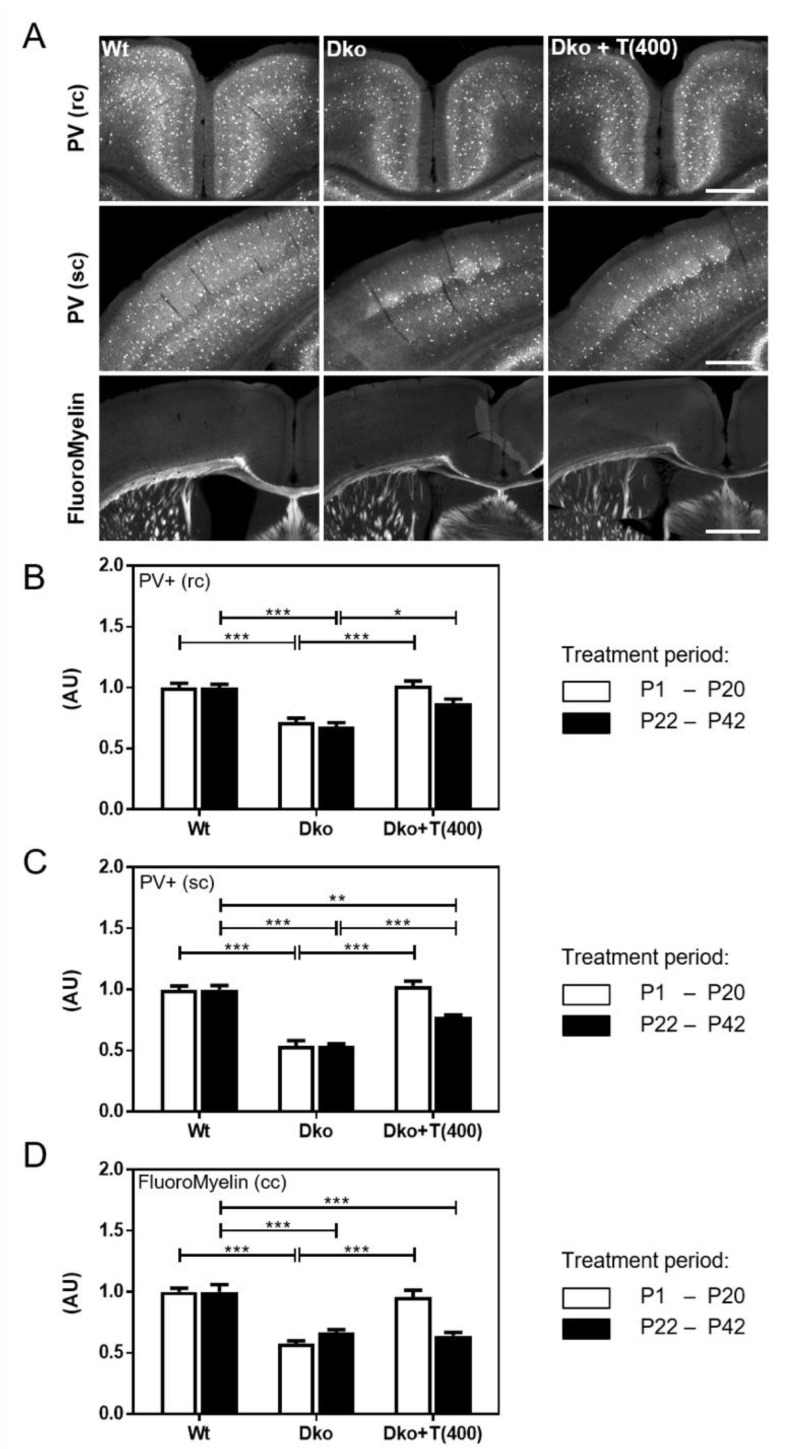
Delayed onset of Triac application compromises efficacy of the treatment. Dko mice were injected with saline or 400 ng/g bw Triac either between P1 and P20 or P22–P42 and analyzed at P43. (**A**) PV+ interneurons were visualized in the retrosplenial cortex (rc) or somatosensory cortex (sc) and myelination was assessed in the corpus callosum (cc) by FluoroMyelin staining. Pictures of animals treated with Triac between P22–P42 are shown. Low numbers of PV+ neurons (normalized to respective Wt numbers) in saline-injected Dko mice were fully restored following early onset Triac treatment while upon late onset only a partial recovery was observed in the rc (**B**) and sc (**C**,**D**) FluoroMyelin integrated density in the cc indicated rescue of Dko hypomyelination phenotype by Triac application between P1–P20 only. n = 3–4. Scale bars 250 µm (PV), 500 µm (FluoroMyelin). *, *p* < 0.05; **, *p* < 0.01; ***, *p* < 0.001.

Obviously, high-dose Triac application restored myelination and improved maturation and function of the GABAergic system when applied between P1 and P20. In order to determine the critical time window of Triac action, we repeated our studies with a second cohort of Dko mice that only received Triac treatment between postnatal days P22–P42 (Figure 6). Only Dko mice that received Triac during the first 3 postnatal weeks showed a full normalization of PV+ cell numbers in retrosplenial (Figure 6B) and somatosensory cortex (Figure 6C) as well as normal myelination in the corpus callosum assessed by FluoroMyelin (Figure 6D). In contrast, in Dko mice that received Triac only between postnatal week 3 and 6, myelination was not improved while a partial recovery of PV+ immunoreactivity could still be detected. Altogether, our data indicate that the most pronounced beneficial effects of Triac on different brain parameters of Dko mice can only be achieved if postnatal treatment is initiated as early as possible.

## 3. Discussion

AHDS represents a devastating neurodevelopmental disease for which no therapy is currently registered. Early treatment strategies such as the application of T4 in combination with PTU in order to block T4 to T3 conversion aimed to normalize serum TH parameters and to ameliorate the symptoms of peripheral thyrotoxicosis [22]. Such a treatment regiment, however, will not improve neurological symptoms given that a diminished TH transport across brain barriers and thus, a reduced TH action inside the CNS represent the major pathogenic mechanisms. This uptake blockage may be circumvented by applying thyromimetic substances that bypass MCT8 and are able to activate TH receptors in neural target cells. In a compassionate study, Ditpa was given for 26–40 months to four affected children at the age of 8–25 months and was able to both normalize high serum T3 concentrations and reduce symptoms of hypermetabolism [18]. Improvements in neurological functions, however, have not been reported. Likewise, in a first international clinical trial with 46 AHDS patients (median age 7.1 years) which were treated with Triac for 12 months, a rapid reduction of the high serum T3 levels as well as beneficial and sustainable effects regarding clinical signs of a peripheral thyrotoxicosis could be observed [21]. This Triac trial was not designed to detect any impact of the treatment on neurodevelopmental outcomes in human MCT8 deficiency although a trend towards neurodevelopmental improvement was noted in a subset of patients in an exploratory analysis. Thus, it is still an open question whether Triac (and Ditpa) treatment has any long-lasting beneficial effects on CNS development. A second clinical phase 2 study, Triac trial II (NCT02396459), has recently been initiated to specifically assess Triac effects in patients younger than 30 months of age at the start of the treatment; whereas, a similar clinical trial for Ditpa is in preparation (NCT04143295).

Although both TH analogs have already been tested in different model systems [15,16,19,23,24,25,26,27], previous studies did not allow to draw any conclusions as to which of the two compounds exerts stronger beneficial effects on CNS development due to a variety of confounding factors (i.e., usage of different Mct8 mutants, treatment regimens, read-out parameters, and drug concentrations). The major aim of our study was, therefore, to evaluate and directly compare the thyromimetic potential of Ditpa and Triac in Mct8/Oatp1c1 double knock-out (Dko) mice as a preclinical AHDS model. Due to a profound CNS-specific TH deprivation, these Dko mice exhibit distinct brain histomorphological abnormalities (hypomyelination, compromised cerebral, and cerebellar neuronal differentiation) that were also seen in AHDS patients [6,7,13]. Moreover, by detecting a rise in cortical mIPSC frequencies in Dko mice (Figure 5), we provide another relevant read-out parameter that underscores the impact of Mct8/Oatp1c1 double-deficiency on inhibitory neuronal network activity.

Here, we provide a first systematical testing of different concentrations of Triac and Ditpa in parallel. Two doses from previous concentration-finding pilot studies were employed for Triac [16]. For Ditpa, we used 10-fold higher concentrations compared to Triac as such an order-of-magnitude-increase was needed to achieve similar thyromimetic effects in cerebellar Purkinje cell outgrowth in vitro (not published). Likewise, 10-times higher concentration of Ditpa compared to Triac were needed to induce myelin marker p0 mRNA expression in mct8 mutant zebrafish to a similar extent [25]. Moreover, we focused on brain parameters as read-outs that are well-established TH targets and that have been reported to be affected in AHDS patients as well as in our AHDS mouse model of Mct8/Oatp1c1 double-deficiency [7,13].

As major findings of our study, we could demonstrate for the first time robust and long-lasting beneficial effects of Triac on various brain parameters in Dko mice only if a high-dose Triac treatment was initiated directly after birth. This regimen was sufficient to fully restore cerebellar development and myelination as well as maturation of cortical GABAergic neurons in Dko mice as evidenced by immunofluorescence analysis (Figure 1). Cortical mIPSC frequencies recorded in acute brain slices were elevated in Dko animals and showed normal values in high-dose Triac-treated Dko animals (Figure 5). Possibly, these electrophysiological findings reflect the altered expression pattern of PV and other calcium-binding proteins in Dko mice as a reduced neuronal Ca^2+^ buffering capacity will cause an elevated release probability and thus, a rise in mIPSC frequency.

Finally, high-dose Triac treatment of Dko mice restored locomotor performance as assessed by rotarod and hanging wire test (Figure 3). In comparison, high-dose Ditpa treatment during the first three postnatal weeks also stimulated myelination and cerebellar development but was significantly less effective in mending cortical GABAergic neuron maturation and normalizing locomotor function. Although we currently cannot rule out that the application of an even higher dose of Ditpa (>4000 ng/g bw) would be as effective as Triac in restoring brain maturation in Dko mice, our preclinical findings suggest a stronger potential for Triac for the treatment of MCT8 patients.

Several aspects, however, must be considered before translating these preclinical data into clinical practice. The high concentration of Triac needed for effective normalization of brain parameters in our mouse model resulted in a fully downregulated HPT axis with *Tshb* transcript below the detection limit (Figure 2A,B). Although we were not able to determine serum Triac versus TH concentrations in Triac-treated animals due to antibody cross-reactivity issues [28] we speculate that endogenous serum TH concentrations are highly reduced in Triac-treated Dko mice. Triac is most likely the only TH receptor active substance in these animals with such a high thyromimetic activity (at the highest dose) that it still causes a peripheral thyrotoxic state as indicated by the elevated hepatic and renal *Dio1* expression (Figure 2D). This scenario is certainly in contrast to the situation of the AHDS patients in the first clinical Triac trial [21]. In fact, one major aim of this clinical study was to achieve a reduction in the highly elevated serum T3 levels in order to ameliorate peripheral thyrotoxicosis while T4 serum levels should still be detectable. To that end, an average dose of 38.3 µg/kg/day was given, which is in the same range as the low Triac dose (T (50)) used in this study. However, treating Dko mice with such a low Triac dose had only little beneficial effects on brain parameters such as myelination or interneuron maturation (Figure 1). It is, therefore, tempting to speculate that with respect to the neurological outcome, MCT8 patients might benefit more from a treatment with a higher dose of Triac even if under these circumstances signs of e.g., hepatic thyrotoxicosis are still present.

Another intriguing observation is the optimal time window of Triac application. In our study, only the early onset Triac treatment regiments exerted strong beneficial effects on brain parameters in Dko while late onset Triac application starting at postnatal day 22 and lasting for 3 weeks did not improve myelination and only insufficiently restored cortical PV+ cell numbers (Figure 6). Why the CNS of Dko mice showed very little response to the late-onset Triac treatment is still elusive. As one hypothesis, the not-yet identified transport systems by which Triac enters the CNS and neural target cells might be down-regulated in the mature mouse CNS [23]. Additionally, the critical time window during which neural differentiation processes are still sensitive to TH-analog treatment might be closed. The latter speculation is based on clinical observations of LT4-treated congenital hypothyroid patients who benefit the most from a neonatal commencement of L-T4 substitution. In contrast to congenital hypothyroidism, MCT8 deficiency is associated with brain abnormalities already visible at fetal stages [7]. Thus, any TH-analog treatment, even if initiated directly at birth, might ameliorate distinct brain parameters to a lesser extent in AHDS patients compared to Dko mice. Despite these limitations, our preclinical findings underscore the utmost importance of an early postnatal diagnosis of AHDS followed by an immediate treatment initiation.

As an alternative to TH-analog treatment, gene therapy approaches exploiting AAV vector constructs have been considered [29,30,31]. These interventions aim to express a functional MCT8 transporter in brain endothelial cells and eventually restore TH action in other neural cell types. Intravenous injection of endothelial cell specific AAV-BR1-Mct8 constructs in newborn Dko mice resulted in improved cerebellar development, myelination, and GABAergic-marker expression [31]. Yet, these beneficial effects were less profound compared to the alterations seen here upon high-dose Triac treatment in Dko mice. Treatment of juvenile Dko mice with AAV-BR1-Mct8 at P30 induced expression of well-established T3-target genes yet failed to improve myelination and only slightly induced GABAergic-marker expression, similar to our observations with late onset Triac treatment. Likewise, in Dko mice treated at P30 with AAV9-MCT8 constructs, serum TH parameters remained abnormal indicating that a peripheral hyperthyroidism is preserved, while TH-target genes in the CNS showed only a partial response [30].

By comparing different treatment strategies and similar read-out parameters in the same Dko mouse model, Triac appears as the most promising treatment approach for AHDS to date. Yet, Triac presumably needs to be given at a high dose and the treatment should be initiated as early as possible to achieve the best outcome for the patients. It also remains to be considered that compared to mice, human brain development is more advanced at birth and thus, even a neonatal initiation of Triac in AHDS patients may not lead to such profound neural improvement as seen in Dko mice.

## 4. Materials and Methods

### 4.1. Animals

Generation and genotyping of mice lacking concomitantly Mct8 (Slc16a2^tm1Dgen^; MGI: 3710233) and the organic anion transporting protein Oatp1c1 (Slco1c1^tm1.1Arte^; MGI: 5308446) were reported elsewhere [8,11,13]. Heterozygous breeding pairs on C57Bl/6N background were set up to obtain Mct8/Oatp1c1 double knock-out (Dko) offspring and wildtype (Wt) animals that served as controls [13]. All animals were supplied with regular chow and water ad libitum and housed in IVC cages at a constant temperature (22 °C) and light cycle (12 h light, 12 h dark). Since the Mct8 encoding gene Slc16a2 is encoded on the X-chromosome, only male mice were used in this study.

Animals were daily s.c. injected between 3–5 pm in the afternoon with TH analogs for the indicated periods. Triac (3,5,3′-L-triioothyroacetic acid; T7650; Sigma-Aldrich, St. Louis, MO, USA) and Ditpa (3,5-diiodo-thyropropionic acid; D4317; Sigma-Aldrich) were dissolved at a concentration of 4 mg/mL and 40 mg/mL in 0.1 N NaOH, respectively, and stored in aliquots at −20 °C. Upon usage, the respective stock solution was diluted in saline. Mice received 2 µL/g body weight (bw) of the desired drug concentrations (Triac: 50 ng/g bw (T (50)) and 400 ng/g bw (T (400)); Ditpa: 400 ng/g bw (D (400)) and 4000 ng/g bw (D (4000)). Control mice received saline injections (2 µL/g bw).

For immunohistochemical analysis, animals were intracardially perfused with 4% paraformaldehyde (PFA) in PBS and brains were post-fixed over-night. Coronal forebrain and sagittal cerebellar sections (50 µm) were produced with a vibratome (ThermoFisher Scientific, Waltham, MA, USA). Animals intended for in situ hybridization studies (ISH), quantitative PCR analysis (qPCR), and TH measurements were killed by CO_2_ inhalation. For ISH, tissues were snap-frozen in 2-methylbutane on dry-ice and stored at −80 °C until further processing. Coronal forebrain cryo-sections (20 µm) and pituitary sections (14 µm) were produced with a cryostat (Leica Biosystems, Nussloch, Germany), mounted on superfrost plus slides and stored at −80 °C. For qPCR analyses, tissue was frozen on dry ice and stored at −80 °C. Serum was obtained from whole blood samples collected by cardiac puncture using microvette tubes (Sarstedt; Nümbrecht, Germany) and stored at −20 °C. Serum TH concentrations were determined as described elsewhere [32,33].

### 4.2. Immunohistochemistry

Staining and analysis was carried out as detailed elsewhere [13]. In brief, mid-sagittal cerebellar vibratome sections were blocked and permeabilized with 10% normal goat serum in PBS containing 0.2% Triton X-100 and immunostained with a mouse anti-calbindin D28k antibody (Sigma-Aldrich, 1:1000) followed by incubation with Alexa Fluor 555-labeled goat anti-mouse secondary antibody (Thermo Fisher Scientific, 1:1000).

Coronal forebrain sections were blocked and permeabilized as above, stained with rat anti-MBP (Merck Millipore, Burlington, VT, USA, 1:300), mouse anti-PV (Millipore, 1:1000), or mouse anti-GAD67 (Millipore, 1:200), and incubated with Alexa Fluor 555-labeled secondary antibody produced in goat (Thermo Fisher Scientific, all 1:1000). To visualize myelin, sections were incubated with FluoroMyelin Green Stain according to the manufacturer’s instructions (Molecular Probes, Eugene, OR, USA, 1:300 dye dilution). Sections between Bregma 1.045 and −1.555 were employed for these analyses.

Pictures were taken with an Olympus AX70 microscope or Zeiss ApoTome2. For quantification of the Purkinje cell (PC) outgrowth, thickness of the molecular layer (ML) that reflects the dimension of the PC dendritic tree was determined at three different positions in lobules III, IV and V using ImageJ. PV positive neurons were counted in all layers of the somatosensory and retrosplenial cortex and normalized to the size of the analyzed area. For quantifying MBP, GAD67, and FluoroMyelin staining intensities, the respective integrated fluorescence signal intensities per area were measured using ImageJ software. Wt average values were set as 1.0. Blinding was achieved by attributing random numbers to the pictures. For each analysis, four brain sections per animal from 3–5 mice per experimental group were employed.

### 4.3. ISH histochemistry

CDNA fragments complementary to mouse *Trh* (NM_009426.2, nt 1251–1876), mouse *Tshb* (NM_009432.2, nt 190–445), and mouse *Hr* (NM_021877.2, nt 902–1598) were used as templates for in vitro transcription using [^35^S]-UTP (Hartmann Analytic, Braunschweig, Germany) as a substrate. Radioactively labeled cRNA probes were purified and diluted in hybridization buffer (50% formamide, 10% dextrane sulfate, 0.6 M NaCl, 10 mM Tris-HCl pH 7.5, 1× Denhardt’s solution, 100 µg/mL sonicated salmon sperm DNA, 1 mM EDTA, 10 mM dithiothreitol (DTT) and 0.5 mg/mL t-RNA) to a final concentration of 1 × 10^4^ cpm/µL. In order to prevent overexposure of the ISH signal, the radioactively labeled cRNA probes for *Trh* and *Tshb* were further diluted with the respective unlabeled cRNA probes (5 ng/µL in hybridization buffer) at a ratio of 1:10 (for *Trh*) and 1:3 (for *Tshb*).

ISH was performed according to the hybridization procedures described in detail previously [13]. For detection of radioactive ISH signals dehydrated sections were dipped in Kodak NTB nuclear emulsion and stored at 4 °C for 3 days (*Trh* and *Tshb*) and 8 days (*Hr*). Autoradiograms were developed and analyzed under darkfield illumination with an Olympus microscope. For quantification of ISH signals, 4–6 tissue sections of 4–5 animals per experimental group were analyzed using ImageJ to determine the integrated signal intensities as described previously [13]. Experiments carried out using the respective sense cRNA probes did not produce any specific ISH signals.

### 4.4. QPCR

Total tissue RNA was isolated using the NucleoSpin RNA II Kit (Macherey-Nagel, Düren, Germany). cDNA synthesis was performed by reverse transcription using the Transcriptor High Fidelity cDNA-Synthesis Kit (Roche, Mannheim, Germany) according to the manufacture’s protocol. For each replicate, 5 ng of cDNA were employed. To exclude the presence of genomic DNA, one sample without reverse transcriptase was included as well. qPCR was performed using the iQ SYBR Green SupermixTM (Bio-Rad; Hercules, CA, USA) and a Bio-Rad CFX384 detection system. Following primers were used: 

Cyclophillin D: 5′-GCAAGGATGGCA-AGGATTGA-3′ and 5′-AGCAATTCTGCCTGGATAGC-3′; Dio1: 5′-CGTGACTCCTGAAGATGATG-3′ and 5′-CCAATGCCTATGGTTCCTAC-3′. The primer pairs were designed for an annealing temperature of 55 °C. Transcript levels of Dio1 were normalized to expression levels of Cyclophillin D as a house-keeping gene. Four samples per experimental group were subjected to analysis.

### 4.5. Electrophysiology

For electrophysiological recordings, 350-µm-thick brain slices were prepared from 3 week old mice and equilibrated in artificial cerebrospinal fluid (aCSF) composed of 120 mM NaCl, 3 mM KCl, 1.3 mM Mg_2_SO_4_, 1.25 mM NaH_2_PO_4_, 2.5 mM CaCl_2_, 10 mM D-glucose, and 25 mM NaHCO3, and gassed with 95% O_2_/5% CO_2_, pH 7.3 at room temperature for at least 1 h as described previously [34].

Patch clamp recordings were performed on coronal slices that were placed in a submerged recording chamber mounted on an upright microscope (BX51WI, Olympus, Hamburg, Germany). Slices were continuously superfused with gassed aCSF (2–3 mL/min, 32 °C, pH 7.3). Recordings of miniature inhibitory postsynaptic current (mIPSC) kinetics were performed using a CsCl-based intracellular solution containing 122 mM CsCl, 8 mM NaCl, 0.2 mM MgCl_2_, 10 mM HEPES, 2 mM EGTA, 2 MM Mg-ATP, 0.5 mM Na-GTP, 10 mM QX-314 [*N*-(2,6-dimethylphenylcarbamoylmethyl) triethylammonium bromide], pH adjusted to 7.3 with CsOH. DL-AP5 (30 μM), CNQX (10 μM) and tetrodotoxin (0.5 μM) were added to the perfusate. mIPSCs were recorded at a holding potential of −70 mV for at least 5 min in aCSF. Data analysis was performed off-line with the detection threshold levels set to 5 pA. The following parameters were determined: frequency, peak amplitude, rise time, time constant of decay (τ-decay), half-width, and electrical charge transfer.

### 4.6. Behavioral Studies

Motor coordination was evaluated using an Accelerating Rotarod (TSE Systems, Berlin, Germany). Briefly, animals were familiarized to the system by allowing them to run once with a velocity of 5 rpm. On the following day, animals were exposed to acceleration of rod rotation from 5–50 rpm within the maximum testing period of 300 s. The riding time of each animal was recorded twice daily and averaged for five consecutive days. Ten to 14 animals were included in each experimental group.

Muscle strength was assessed in the hanging wire test. Animals were placed on top of a wire cage lid. After shaking gently and turning the cage upside down, the animal’s capability to cling to the wire was monitored. Time was taken until the mouse fell down or else the trial was terminated after 60 s. Each mouse was tested twice daily for three consecutive days and values were averaged. Twelve to 16 mice were used per experimental group.

### 4.7. Statistics

All data are presented as mean ± SD. Comparison between groups was performed by two-way ANOVA for experiments including two doses of each compound while one-way ANOVA analysis was applied for experiments using single doses followed by pairwise Tukey’s post hoc test, if not otherwise indicated. Differences were considered statistically significant if *p* < 0.05.

### 4.8. Study Approval

All animal procedures were in accordance with the European Union (EU) directive 2010/63/EU and approved by the Animal Welfare Committees of the Thüringer Landesamt für Lebensmittelsicherheit und Verbraucherschutz (03-011/11; TLLV; Bad Langensalza, Germany) and the Landesamt für Natur-, Umwelt- und Verbraucherschutz Nordrhein-Westfalen (84-02-04.2015.A331; LANUV; Recklinghausen, Germany).

## Data Availability

The data presented in this study are available on request from the corresponding author.

## Supplementary Materials

The following supporting information can be downloaded at: https://www.mdpi.com/article/10.3390/ijms24043452/s1.
