# Supplementary material for: Triac Treatment Prevents Neurodevelopmental and Locomotor Impairments in Thyroid Hormone Transporter Mct8/Oatp1c1 Deficient Mice
Source: Int J Mol Sci. 2023 Feb 9;24(4):3452. doi: 10.3390/ijms24043452 (PMC9966820; doi:10.3390/ijms24043452)
Supplement: Supplementary file 1 [file ijms-24-03452-s001.zip › ijms-2116104-Supplementary Figure S1.pdf]

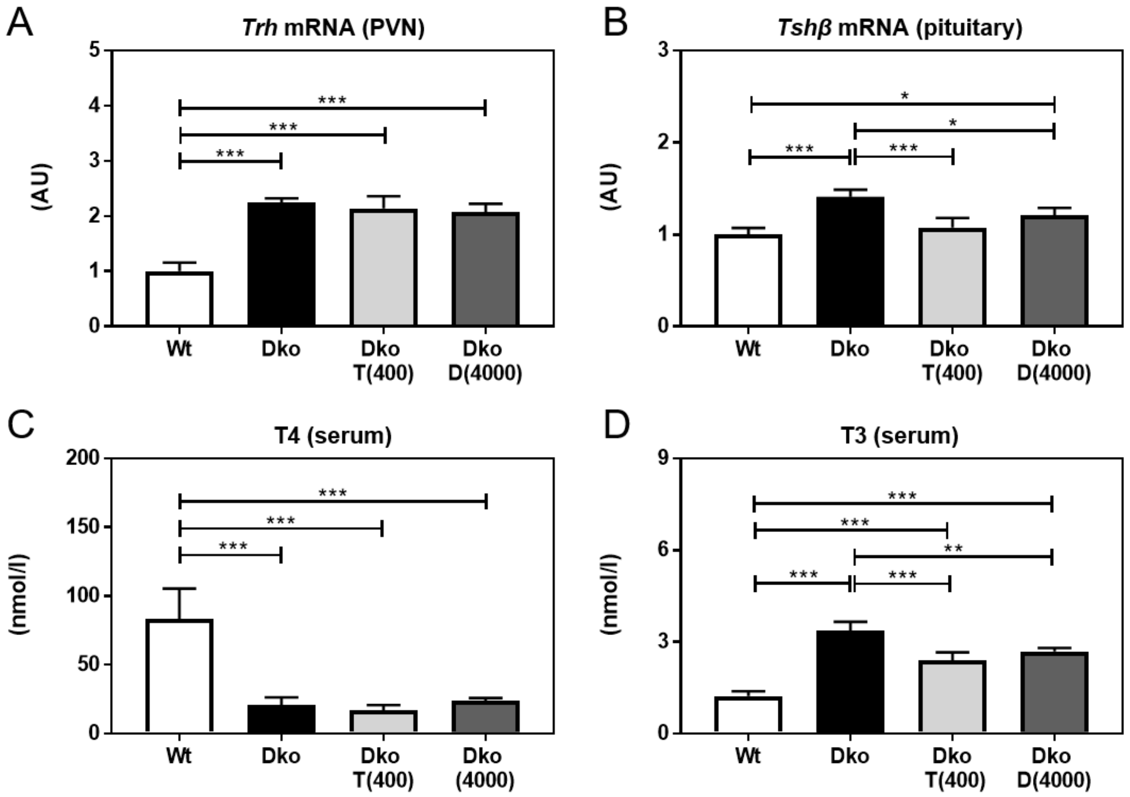

Supplementary Figure S1: Early postnatal TH analog application permanently modulates the set point of the HPT axis. Activity of the HPT axis was monitored at 10 weeks of age in animals receiving TH analogs Triac or Ditpa daily between P0 and P20 only. A) Radioactive ISH was performed and *Trh* mRNA expression in the PVN and levels of Tsh beta transcripts in the pituitary were quantified. Following cessation of TH analog treatment, Trh levels returned to abnormally high values as seen in saline-injected Dko mice. In the pituitary, *Tshb* expression was still decreased in animals that received an early, transient treatment with TH analogs. B) TH serum levels were determined. T4 was equally low in all Dko animals independent of the treatment, whereas T3 concentrations significantly reduced in Triac and Ditpa-treated Dko mice in comparison to saline-injected genotype controls. n=3-5; *, $p < 0.05$; **, $p < 0.01$; ***, $p < 0.001$.
